# Defining the Pharmacodynamic Profile and Therapeutic Index of NHS-IL12 Immunocytokine in Dogs with Malignant Melanoma

**DOI:** 10.1371/journal.pone.0129954

**Published:** 2015-06-19

**Authors:** Melissa Paoloni, Christina Mazcko, Kimberly Selting, Susan Lana, Lisa Barber, Jeffrey Phillips, Katherine Skorupski, David Vail, Heather Wilson, Barbara Biller, Anne Avery, Matti Kiupel, Amy LeBlanc, Anna Bernhardt, Beatrice Brunkhorst, Robert Tighe, Chand Khanna

**Affiliations:** 1 Comparative Oncology Program, Center for Cancer Research, National Cancer Institute, Bethesda, Maryland, United States of America; 2 College of Veterinary Medicine, University of Missouri-Columbia, Columbia, Missouri, United States of America; 3 College of Veterinary Medicine and Biological Sciences, Colorado State University, Fort Collins, Colorado, United States of America; 4 School of Veterinary Medicine, Tufts University, North Grafton, Massachusetts, United States of America; 5 College of Veterinary Medicine, University of Tennessee, Knoxville, Tennessee, United States of America; 6 School of Veterinary Medicine, University of California Davis, Davis, California, United States of America; 7 School of Veterinary Medicine, University of Wisconsin-Madison, Madison, Wisconsin, United States of America; 8 College of Veterinary Medicine, Texas A&M University, College Station, Texas, United States of America; 9 College of Veterinary Medicine, Michigan State University, East Lansing, Michigan, United States of America; 10 EMD-Serono Research and Development Institute, Billerica, Massachusetts, United States of America; University of Pittsburgh, UNITED STATES

## Abstract

**Background:**

Interleukin (IL)-12 is a pro-inflammatory cytokine that mediates T-helper type 1 responses and cytotoxic T-cell activation, contributing to its utility as anti-cancer agent. Systemic administration of IL-12 often results in unacceptable toxicity; therefore, strategies to direct delivery of IL-12 to tumors are under investigation. The objective of this study was to assist the preclinical development of NHS-IL12, an immunocytokine consisting of an antibody, which targets necrotic tumor regions, linked to IL-12. Specifically this study sought to evaluate the safety, serum pharmacokinetics, anti-tumor activity, and immune modulation of NHS-IL12 in dogs with naturally occurring cancers.

**Methodology/Principal Findings:**

A rapid dose-escalation study of NHS-IL12 administered subcutaneously to dogs with melanoma was conducted through the Comparative Oncology Trials Consortium (COTC). Eleven dogs were enrolled in four dose-escalation cohorts; thereafter, an additional seven dogs were treated at the defined tolerable dose of 0.8 mg/m^2^. The expanded cohort at this fixed dose (ten dogs in total) was accrued for further pharmacokinetics and pharmacodynamics assessment. NHS-IL12 levels, serum cytokine concentrations, and peripheral blood mononuclear cell characterization (post-treatment) and draining lymph node immune profiling, and tumor biopsies (pre- and post-treatment) were collected. Adverse events included thrombocytopenia, liver enzymopathies, fever, and vasculitis. Correlation between interferon (IFN)-γ induction, adverse events, and NHS-IL12 exposure (maximum concentration and area under the concentration-time curve) were dose-dependent. Serum IL-10 levels and intratumoral CD8^+^ populations increased after treatment. Partial responses, according to Response Evaluation Criteria in Solid Tumors (RECIST) criteria, were observed in two dogs treated with NHS-IL12 0.8 mg/m^2^ and 1.6 mg/m^2^.

**Conclusions/Significance:**

NHS-IL12 was administered safely to dogs with melanoma and both immunologic and clinical activity was observed. This study successfully defined a narrow therapeutic window for systemic delivery of NHS-IL12 via the subcutaneous route. Results will inform the design and implementation of first-in-human clinical trials of NHS-IL12 in cancer patients.

## Introduction

The opportunity to treat cancer patients effectively by augmenting or directing anti-tumor immune responses (i.e. immunotherapy) has increasingly expanded into the clinical arena. The use of cytokines to stimulate immune responses against cancer has been recognized as an important component of immunotherapy. Interleukin (IL)-2, tumor necrosis factor (TNF)-α, interferon (IFN)-α, IL-6, IL-18, and IL-12 are in various stages of development [1–8] Indeed, the use of IL-2 as a cytokine-based immunotherapy was first used in human patients in 1985, and was approved in patients with melanoma in 1998 based on durable, but low, complete response rates (< 10%) [9]. Among potential cytokines, interest in IL-12 is based on its central role in bridging innate and adaptive immune responses. IL-12 mediates its immune function by generating T-helper type 1 responses and has shown anti-cancer activity in renal cell carcinoma, cutaneous T-cell lymphoma, and melanoma [10]. It induces proliferation and activation of natural killer and T cells to enhance tumor immunity. In addition, IL-12 inhibits tumor angiogenesis mainly through IFN-γ-dependent production of the chemokine IP-10 [11]. The clinical development of IL-12 has been hindered by unpredictable and sporadic response rates and unacceptable toxicity [8,12]. Safety concerns surrounding acute and chronic toxicities, as well as the reliable prediction of which patients will experience adverse events, remain problematic for its development [13]. Therefore, strategies to target the delivery of IL-12 to tumors rather than to normal tissues are needed and are currently being investigated.

NHS-IL12 is a fusion protein comprising two IL-12 heterodimers combined to the H-chain of an NHS76 monoclonal antibody. NHS76 binds to DNA/histone complexes, thereby targeting IL-12 to regions of tumor necrosis where DNA is exposed. Because necrosis is a universal feature of solid malignancies during tumor growth, NHS76 targeting is thought to have broad applicability across multiple indications. Murine in-vivo models have shown NHS-IL12 to be safe and efficacious with a subcutaneous route of administration showing the best results. This route of administration achieves a slow, sustained release into the bloodstream with enhanced lymphatic absorption to potentiate immune responses in loco-regional lymph nodes prior to systemic distribution. However, a greater understanding of the optimal dose, schedule, pharmacokinetics, and immunological changes linked to drug exposure are needed to translate NHS-IL12 into human clinical trials.

Comparative oncology provides an opportunity for translational studies of new cancer therapeutics using pet dogs that have developed cancer naturally [14,15]. Studies in tumor-bearing dogs can answer questions about a drug’s pharmacokinetics, mechanism of action, pharmacodynamic modulation, and can define more specifically same species dose-toxicity and dose-response relationships in ways that are difficult to assess in mouse models and human trials [16]. Comparative oncology studies of immunotherapeutics are particularly informative due to the presence of a syngeneic host-tumor relationship and a competent immune system. Other advantages of the dog model are opportunities to serially biopsy naturally-occurring malignant lesions during a course of therapy, and assess constitutional signs of fatigue or lethargy, which can be difficult and/or impossible in rodent studies. Tumor vaccines, liposome cytokine delivery, immunomodulators, and other immunotherapeutics have been evaluated successfully in dogs with cancer [17–20].

With these comparative- and translational-drug development questions in mind, a clinical trial to evaluate NHS-IL12 administered subcutaneously was conducted with the aim of defining its tolerability and immunologic activity in a spontaneous cancer model. The study design included a fixed-schedule, dose-escalation phase followed by an expanded cohort in dogs with malignant melanoma. Pharmacokinetic analysis and pharmacodynamic endpoints, including serum IFN-γ cytokine concentrations and evaluation of changes in both systemic and tumor-associated immune-cell subsets following NHS-IL12 exposure, were assessed. Serum IL-10 concentrations and intratumoral CD8^+^ populations increased after NHS-IL12 treatment and were measurable biomarkers of exposure. Results from this and other comparative studies will inform the design and implementation of first-in-human clinical trials by defining starting doses of NHS-IL12 and biomarkers for its exposure.

## Results

### Study design and patient characteristics

A fixed schedule, dose-escalation (28-day cycle) study design was used to assess the tolerability of NHS-IL12 administered subcutaneously in dogs with melanoma. This was followed by a fixed-dose, expanded cohort to refine the exposure-biological response relationships in the same population. Dose-escalation cohorts initially consisted of one dog per cohort. A rapid escalation was planned through five dose cohorts (NHS-IL12, 0.4, 0.8, 1.2, 2.4, 3.6 mg/m^2^ subcutaneously) based on adverse events using modified Veterinary Cooperative Oncology Group Common Terminology Criteria for Adverse Events (modified VCOG-CTCAE) [21]. Cohorts were expanded to include three dogs if a grade 2 adverse event was observed or six dogs if dose-limiting toxicity (DLT; defined in Methods) was observed. A total of 11 dogs were enrolled in the dose-escalation phase: 0.4 mg/m² (n = 1), 0.8 mg/m² (n = 3), 1.6 mg/m² (n = 4), 2.4 mg/m² (n = 3). An additional seven dogs were enrolled in the fixed-dose (NHS-IL12, 0.8 mg/m² subcutaneously) expanded cohort. NHS-IL12 treatment was administered on day 1 and serial serum pharmacokinetics and pharmacodynamics (IFN-γ, cytokine profiling, peripheral blood mononuclear cell [PBMC] immune cell characterization) collections were obtained over the first 48 hours, and then repeated as indicated in Tables 1 and 2. Tumor biopsies for evaluation of immune cell infiltrates were collected pre- and post-treatment (days 8 and 29, and monthly thereafter). Response was assessed on day 29 using Response Evaluation Criteria in Solid Tumors (RECIST) criteria [22,23]. Immunogenicity (anti-NHS-IL12 antibodies) was measured in all dogs. Dogs had naïve or recurrent malignant melanoma (although one dog was later reclassified as having an anaplastic sarcoma) and were required to have tumors located peripherally to allow access for biopsy. Sex (six castrated males, one intact male, 11 spayed females), body surface area (range 0.34–1.30 m^2^), and breed (six mixed breed and 12 purebred) were recorded for all dogs in the study. Metastatic disease was evident at enrollment in 11/18 treated dogs.

**Table 1:** Study schedule for dose escalation and cohort expansion of NHS-IL12 administered subcutaneously.

Action	Pre-Tx	Day 1	Day 2	Day 3	Day 8	Day 15	Day 22	Day 29	q. Day 8	q. 28 days
**Patient eligibility**	X									
**Tumor measurements**	X				X	X	X	X	X	X
**Tumor biopsy**	X				X			X		X
**Draining lymph node fine-needle aspirates**	X				X			X		X
**Serum (IFN-γ, cytokines, drug levels, immunogenicity)**		X	X	X	X	X		X	X	X
**PBMC immune cell characterization**		X	X	X	X			X		X
**CBC/chemistry and coagulation profiles/UA**		X			X	X		X	X	X
**Abdominal ultrasound/thoracic radiographs**	X							X		X
**NHS-IL12 subcutaneous**		X						X		X
**Weight measurement**	X		X		X	X	X	X	X	X
**Fever monitoring**		X	X				X	X		
**Digital photo**	X				X			X	X	X

CBC: complete blood count; IFN, interferon; PBMC, peripheral blood mononuclear cell; Tx: treatment; UA: urinalysis.

**Table 2:** Serial serum pharmacokinetics and IFN-γ/cytokine panel collections.

Time	0	1 hr	2 hr	4 hr	8 hr	24 hr	32 hr	48 hr	Day 8
Serum collection	X	X	X	X	X	X	X	X	X

IFN: interferon.

### Subcutaneous delivery of NHS-IL12 0.8 mg/m^2^ was well tolerated in dogs with melanoma

In total, 18 dogs were enrolled in the study, with 13 completing the planned 28-day study period. Tolerability was evaluated in all dogs. Adverse events were defined by modified VCOG-CTCAE criteria and reported uniformly across the multicenter Comparative Oncology Trials Consortium (COTC) [21]. DLTs were defined as Grade ≥3 events attributed to drug therapy, excluding transient or non-clinical neutropenia, thrombocytopenia, or hepatic enzymopathy, which were expected events. Adverse events reported that were acute and expected (fever, hepatic enzymopathy, thrombocytopenia, hypotension, vascular leakage syndrome) mirrored those of human patients and normal dogs receiving systemic IL-12 treatment. NHS-IL12 at a dose of 0.8 mg/m² was defined as tolerable in tumor-bearing dogs and was selected for subsequent evaluation in a fixed-dose expanded cohort. No drug-related serious adverse events were reported in dogs treated at this dose (n = 10). Dogs that received NHS-IL12 at doses of 1.6 mg/m² and 2.4 mg/m² experienced Grade 4 or 5 adverse events (n = 4), thus making these doses unacceptable. Events included thrombocytopenia, fever, anorexia, pleural effusion, and severe anemia. Suspected drug-related serious adverse events included systemic vasculitis and/or severe inflammatory response syndrome (SIRS). These results suggest that there is a narrow therapeutic window for NHS-IL12 and additional studies are needed to refine this window further. Non-drug-related serious events were attributed to tumor progression and included seizure activity (2.4mg/m2) and, anorexia (0.8mg/m2) (n = 2).

### Subcutaneous administration of NHS-IL12 resulted in measurable serum drug levels

**Fig 1:**
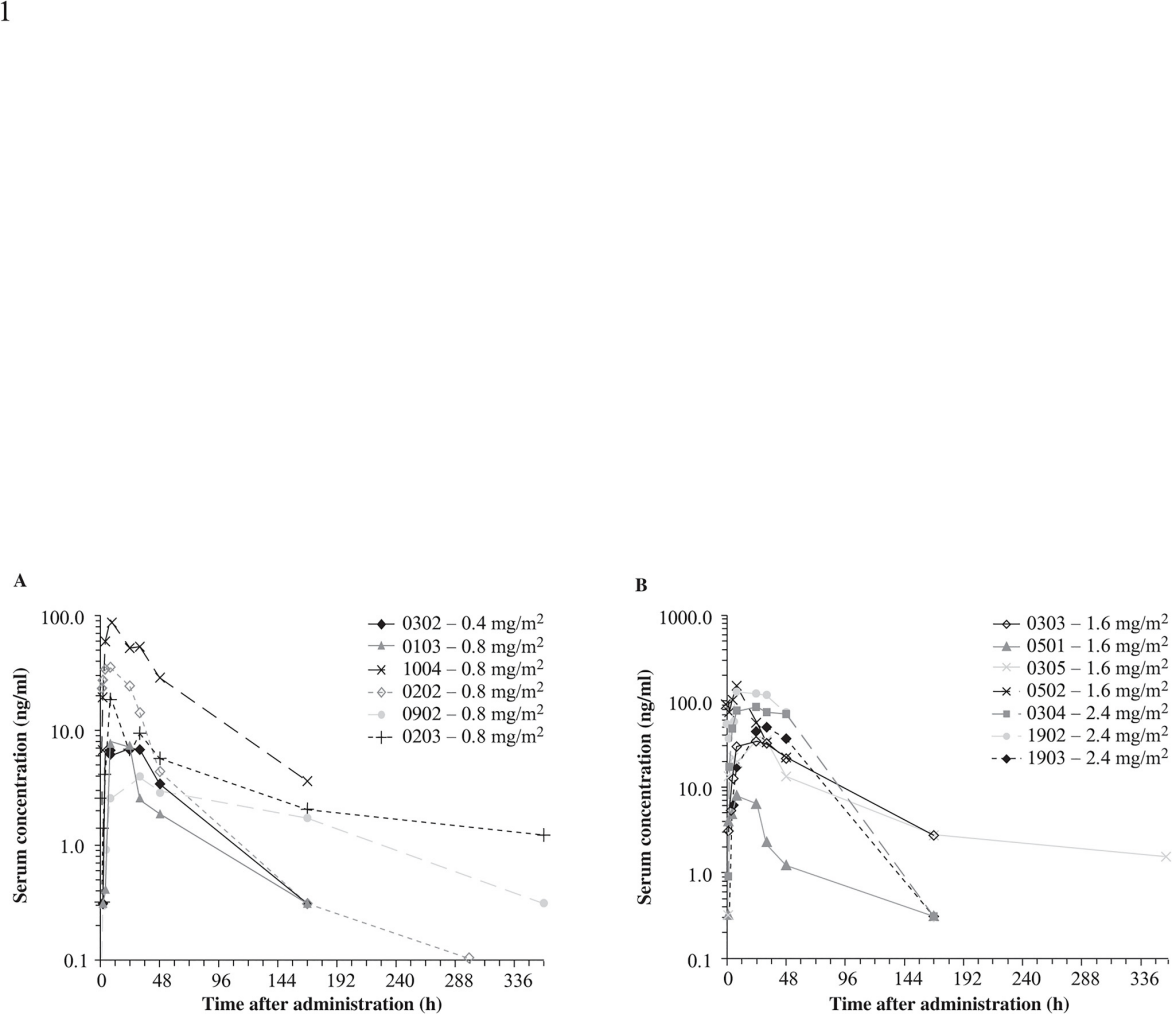
Subcutaneous administration of NHS-IL12 resulted in measurable serum drug levels. Serum samples were collected from dogs to define systemic exposures of NHS-IL12 after subcutaneous administration. NHS-IL12 levels were measured pre-treatment and at 1, 2, 4, 8, 24, 36, and 48 hours following administration (8-point collection) and on days 8, 15, and 29. NHS-IL12 C_max_ was dose-dependent: 0.4 mg/m^2^ and 0.8 mg/m^2^ (A) and 1.6 mg/m^2^ and 2.4 mg/m^2^ (B). Clearance was prolonged in some dogs as NHS-IL12 was still measurable in five animals 14 days following treatment.

Serum samples, collected to assess systemic exposures of NHS-IL12 after subcutaneous administration, were available from 15 dogs and NHS-IL12 levels were measurable in all but two (patients 0201 and 0901; Table 3). Maximum serum concentrations (C_max_) of NHS-IL12 were dose-dependent, although exposure (area under the concentration-time curve [AUC]) was variable across dosing cohorts (Fig 1). Clearance was prolonged in five dogs with NHS-IL12 detectable for up to 15 days following treatment.

**Table 3:** Pharmacokinetics of NHS-IL12 in tumor-bearing dogs after subcutaneous administration.

Animal	Dose (mg/m^2^)	Dose (ug/kg)	V_z_ (mg/kg)	CI (ml/h/kg)	AUC (h*ng/ml)	C_max_(n/gml)	T_1/2_(h)	MRT(h)
**0302**	0.4	20.4	1,820	40.0	497	6.97	31.6	39.0
**0103**	0.8	40.8	1,930	29.9	342	8.02	44.9	37.4
**1004**	0.8	40.8	493	7.86	4,970	91.9	43.5	41.1
**0201**	0.8	40.8	ND	ND	ND	ND	ND	ND
**0202**	0.8	40.8	1,080	31.3	1,290	35.2	23.9	25.6
**0203**	0.8	40.8	4,840	27.2	1,280	20	123	99.4
**0901**	0.8	40.8	ND	ND	ND	ND	ND	ND
**0902**	0.8	40.8	8,130	59.1	648	3.87	95.4	116
**0305**	1.6	81.6	6,440	27.3	2,920	36.0	164	111
**0501**	1.6	81.6	18,900	248	305	7.96	52.6	34.4
**0502**	1.6	81.6	442	21.9	3,290	152	14.0	15.5
**0303**	1.6	81.6	1,560	27.6	2,800	33.8	39.1	44.3
**0304**	2.4	122	1,290	9.01	3,420	85.2	99.2	25.5
**1902**	2.4	122	655	14.9	5,020	129	30.4	23.7
**1903**	2.4	122	NA	NA	1,620	50.3	NA	29.4

AUC: area under the time-concentration curve; Cl: clearance; C_max_: maximum concentration; MRT: mean residence time; NA: not available, calculation not possible due to a small number of data points; ND, not detectable; T_1/2_: half-life.

### Serum cytokine measurements revealed increased systemic IL-10 levels following treatment with NHS-IL12

**Fig 2:**
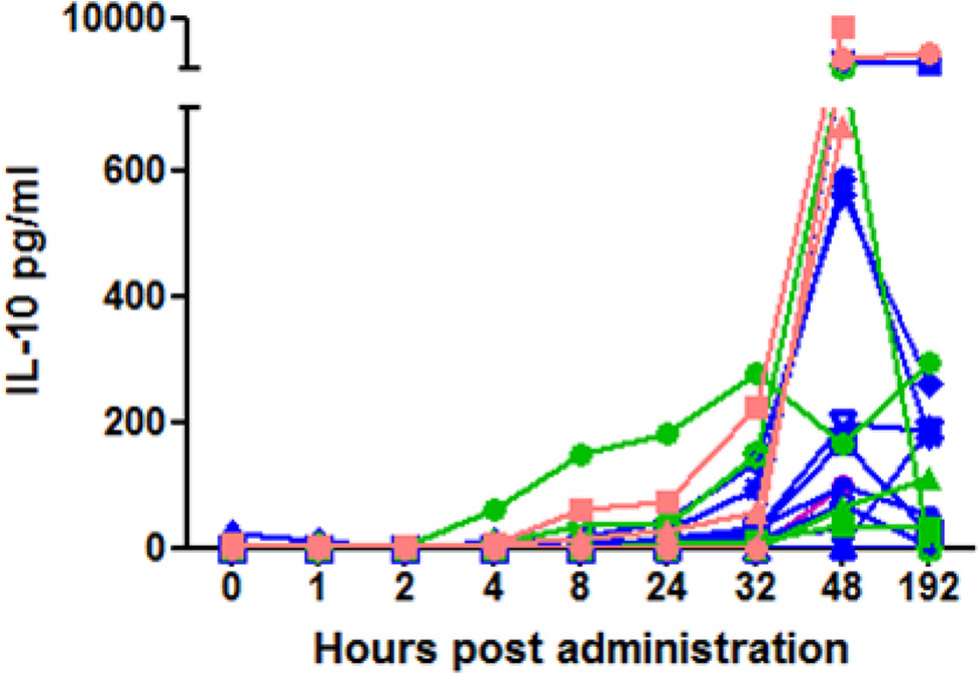
Serum IL-10 levels increased following treatment with NHS-IL12. Each line represents a different dog, with individual colors representing different treatment groups. Serum IL-10 levels at 48 and 192 hours were significantly different from time points 0–8 hours (Kruskal-Wallis test followed by Dunn’s multiple comparison test) when data from all dogs was pooled. There was no difference in IL-10 levels at 48 hours between any of the treatment groups (Kruskal-Wallis test, p = .06, the 0.4 mg/m2 group was not included because it was a single dog).

Canine serum cytokine panels (IL-2, IL-4, IL-6, IL-7, IL-8, IL-10, IL-15, IL-18, Granulocyte-macrophage colony-stimulating factor [GM-CSF], IP-10, keratinocyte-derived cytokine [KC], monocyte chemoattractant protein-1 [MCP-1], TNF-α) were measured before treatment, serially for 48 hours after NHS-IL12 administration, and thereafter as indicated in Table 1. Of these analytes, serum IL-10 levels increased after treatment most consistently (in 17/18 dogs); this increase could be detected as early as four hours after treatment and was statistically significant at both 48 hours and remained so at 192 hours (Fig 2). There was no difference in the levels of serum IL-10 at 48 hours between the three treatment groups with more than one dog. The initial elevation in IL-10 did not correlate with clinical response or with the development of toxicity. There were no other consistent cytokine or chemokine (KC, IL-8, and MCP-1) changes after one cycle of treatment, although IL-8 and MCP-1 were measurable in all dogs. Dramatic increases in cytokines and chemokines (GM-CSF, IL-2, IL-7, IL-15, IL-18, MCP-1) were seen in one dog (patient 0201) after repeated administrations of NHS-IL12. This dog experienced a strong partial response (PR) through nine cycles of treatment.

### Serum IFN-γ induction reflected toxicities at higher NHS-IL12 doses

**Fig 3:**
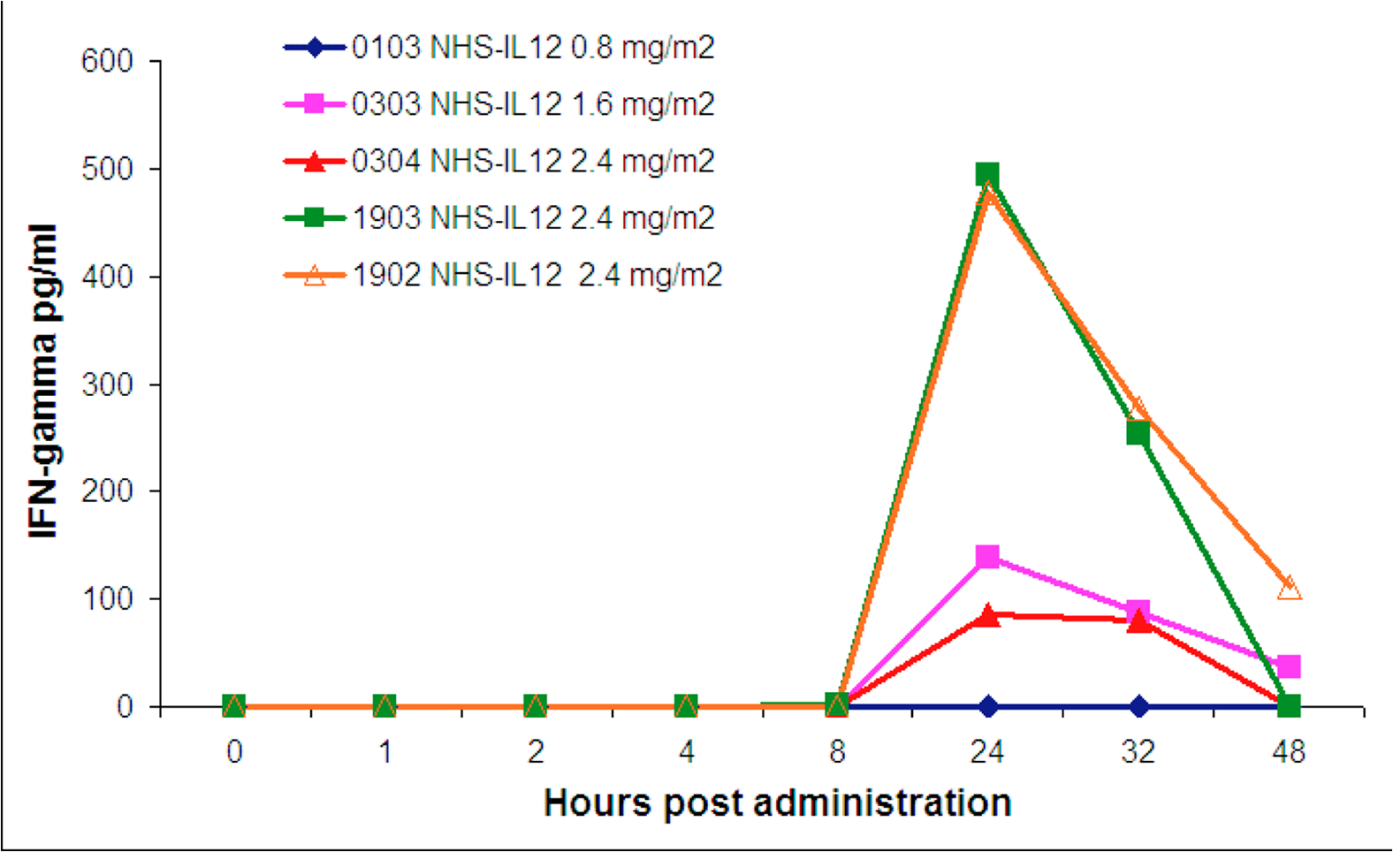
Serum IFN-γ induction was associated with NHS-IL12 dose and the observation of adverse events. IFN-γ levels were measured using ELISA techniques. Induction of IFN-γ was detectable in dogs treated with NHS-IL12 at a dose of 1.6 mg/m^2^ or higher. IFN-γ levels spiked sharply at 24 hours post-treatment and returned to undetectable levels by 48 hours. Elevated serum IFN-γ (> 100 pg/ml) was associated with increased risk for toxicity. However the highest level of IFN-γ induction was not directly linked to the most severe (Grade 4 or 5) adverse events.

Induction of IFN-γ was detectable in dogs that received NHS-IL12 at a dose of 1.6 mg/m^2^ or higher (Fig 3). Elevated serum IFN-γ (> 100 pg/ml) was seen in all dogs experiencing adverse events. However the highest IFN-γ induction was not seen with the most severe (i.e. Grade 4 or 5) events. IFN-γ levels spiked sharply at 24 hours post-administration of NHS-IL12 and returned to undetectable levels by 48 hours. 3/4 dogs treated at 1.6mg/m2 did not have evaluable serum samples.

### Intratumoral CD8^+^ T cell infiltration increased following NHS-IL12 treatment

Tumor histopathology was graded for necrosis, inflammation, and infiltration of T, B, and dendritic cells (CD3^-^CD4^+^ and CD8^+^, CD79a, CD18) pre-treatment and on days 8 and 29 post-treatment. In the dose-escalation phase, intratumoral CD8^+^ T-cell populations increased after NHS-IL12 treatment in 5/7 evaluable dogs. Dogs were deemed evaluable if they had at least two viable tumor biopsies pre- and post-treatment available for immunohistochemical analyses. All dogs with measurable increases in CD8^+^ T cells post-treatment received NHS-IL12 at a dose of 0.8 mg/m^2^ or higher.

### Draining lymph node and PBMC flow cytometry analyses did not reveal distinct trends in immune cell profiling after NHS-IL12 therapy

**Fig 4:**
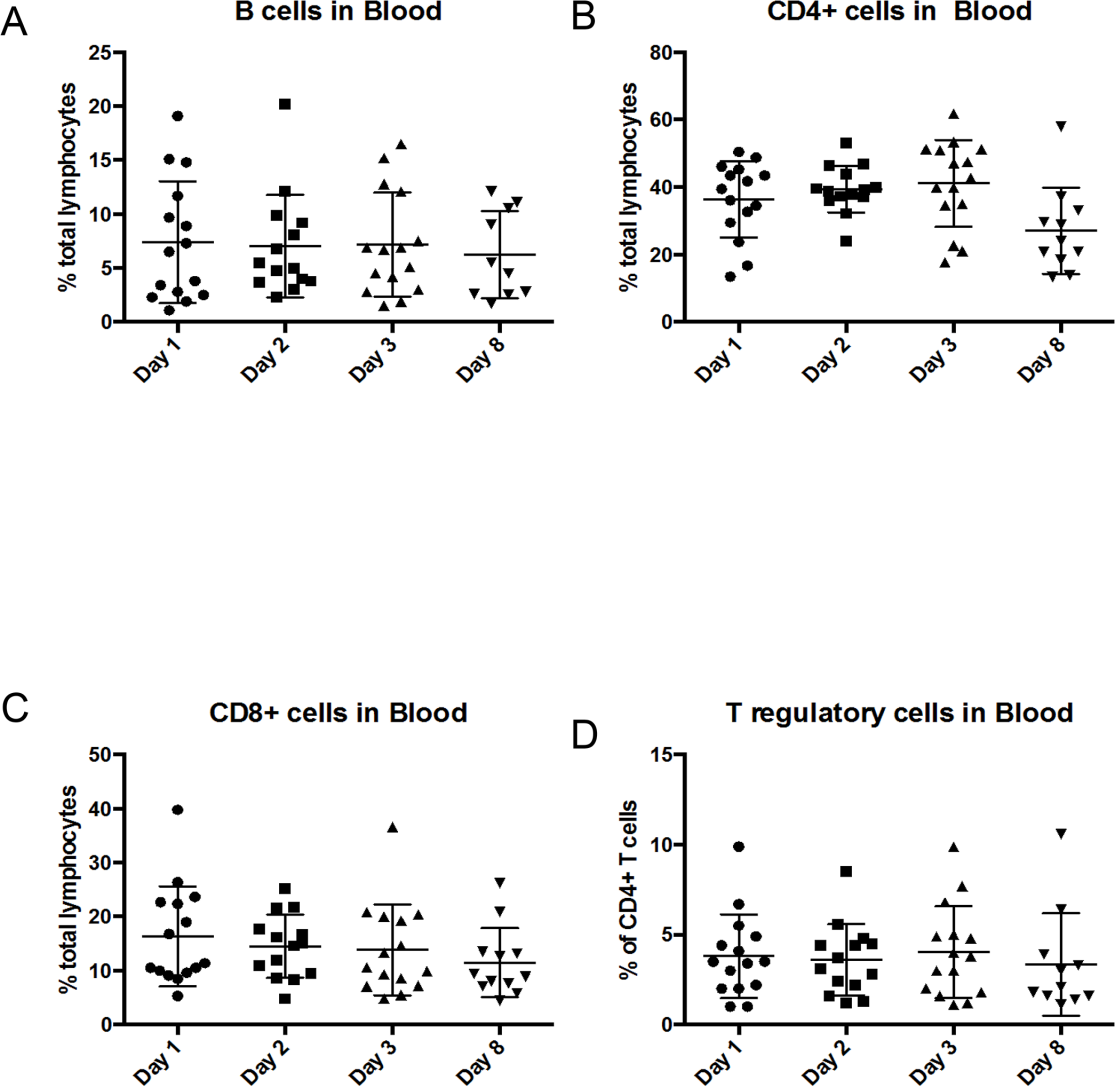
No significant changes in circulating T- or B-cell populations were seen after treatment with NHS-IL12. Relative numbers of T- and B-cell populations were quantified in PBMCs by flow cytometric analysis on days 1, 2, 3, and 8 after treatment with NHS-IL12. Immune cell determination was based on labeling: B cells, CD21^+^MHCII^+^ (A),T cells, CD4^+^ (B) or CD8^+^ (C); regulatory T cells, CD4^+^Foxp3^+^ (D);. CD4^+^ T cells trended downward at day 8 compared to their baseline measurements (B). Other differences were not evident over the time points evaluated.

**Fig 5:**
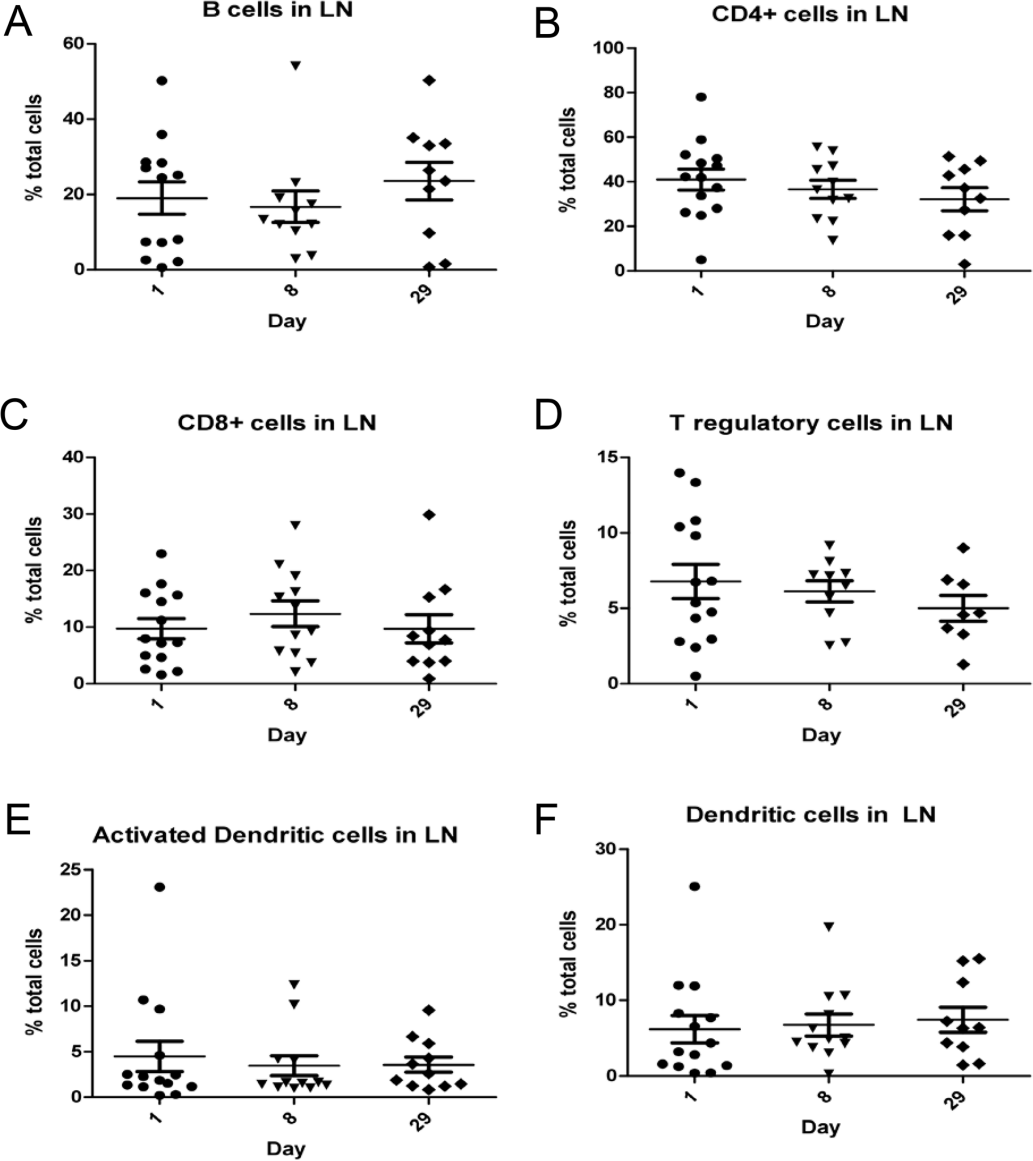
Tumor draining lymph node T-cell, B-cell or dendritic-cell populations did not change significantly after treatment with NHS-IL12. T-cell, B-cell, and dendritic cell populations were quantified in regional lymph node aspirates by flow cytometric analysis on days 1, 8, and 29 after treatment with NHS-IL12. Immune cell determination was based on labeling: B cells, CD21^+^MHCII^+^ (A); T cells, CD4^+^ (B) or CD8+ (C); regulatory T cells, CD4^+^Foxp3^+^ (D); activated dendritic cells, CD11c^+^MHCII^+^ (E); and dendritic cells, CD11c+ (F); No significant differences in subsets were observed for the time points evaluated.

Relative and absolute numbers of T- and B-cell populations were assessed in blood samples obtained on days 1, 2, 3, and 8 post-treatment. Subsets analyzed included CD4^+^, CD8^+^, and CD4^+^Foxp3^+^ T cells (regulatory T cells [Tregs]) and B cells (CD21^+^MHCII^+^). There was a downward trend in CD4^+^ T cells at day 8 post-treatment (Fig 4). The same T- and B-cell subsets (obtained by fine needle aspiration) were analyzed in peripheral tumor draining lymph nodes when readily identifiable on days 1, 8, and 29. Additionally, relative numbers of dendritic cells (CD11c^+^) and activated dendritic cells (CD11c^+^MHCII^+^) were also measured. No significant differences were observed in immune-cell subsets across the time points evaluated (Fig 5).

### Immune responses against NHS-IL12

Production of antibodies to human IL12 was expected in this study as a result of the administration of humanized antibody to dogs. Antibody titers were measured in two dogs on study day 8 and in 12 dogs on study days 15 and 29. As expected, day 8 samples were negative, but 8/12 collected on day 15 or 29 were found to be positive. Host antibody responses were directed mainly against the human IgG part of the molecule, but 5/14 dogs tested also developed antibodies against a huIL12 portion. In 3/14 dogs (patients 0202, 0203, and 0305) negative titers were found for NHS-IL12, human IgG, and both huIL-12 subunits (p40 and p70) four weeks after administration of NHS-IL12. Antibody development did not appear related to the dose of NHS-IL12 administered.

### Clinical activity of systemic NHS-IL12 in dogs with melanoma

**Fig 6:**
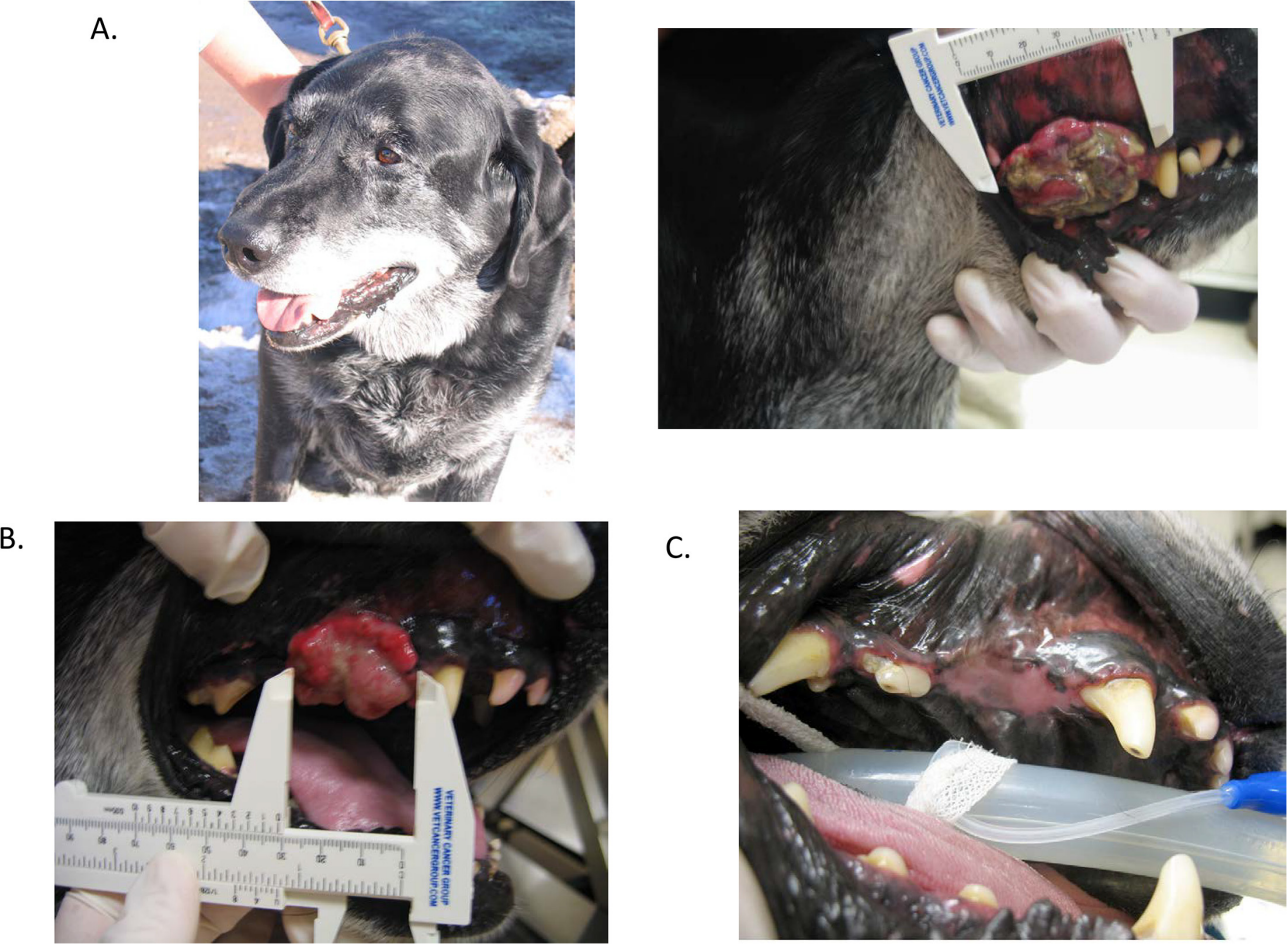
Clinical activity of NHS-IL12 evident in dogs with malignant melanoma. Patient 0201 had a large malignant melanoma of the right maxilla and buccal surface. Prior to treatment on day 1, the tumor measured 4.4 cm in longest diameter (A). The dog received 0.8 mg/m^2^ (1.04 mg) of NHS-IL12 administered subcutaneously. On day 29 RECIST response was assessed and a PR was noted. The tumor measured 2.4 cm, representing a 45% decrease (B). The dog continued monthly dosing of NHS-IL12 and had no evidence of disease after three cycles of therapy (C). On day 85, despite complete regression of the lesion, a formal CR could not be assigned to the observed clinical activity in this dog since canine malignant melanoma invades the underlying bone and a CT scan of the oral cavity was not performed at baseline. No gross disease was noted through nine cycles of therapy. PD eventually recurred in the neighboring nasal cavity.

Tumor target lesions were identified at enrollment and measurements recorded weekly. RECIST criteria were used to evaluate response after one cycle of therapy (28 days) in those dogs achieving an objective response (complete response [CR] or PR) eligible for continued treatment. Overall, 2/7 dogs in the dose escalation that completed one cycle of therapy achieved a PR (NHS-IL12, 0.8 mg/m² [patient 0201] and 1.6 mg/m² [patient 0305]), whereas two dogs had progressive disease (PD) and 3 had stable disease (SD). In addition, patient 0201 (Fig 6) had a prolonged response to treatment and received NHS-IL12 treatment for over 9 months before progression was eventually noted. Four dogs were euthanized prior to day 28, one due to PD and three as a result of drug-related toxicities. In the expanded cohort, seven additional dogs were treated at 0.8 mg/m²; after one cycle of therapy, five had PD and two had SD. Clinical activity recorded at tolerable doses of NHS-IL12 supports its evaluation in further studies.

## Discussion

The clinical utility of cytokines has been hindered by a poor safety profile and incorrectly designed dosing regimens; immunocytokines, such as NHS-IL12, have the potential to overcome these liabilities. The goal of this study was to generate comprehensive data on the tolerability and immunological activity of systemic NHS-IL12 in tumor-bearing dogs. Results show that NHS-IL12 can be safely administered systemically to dogs up to a dose of 0.8 mg/m² and both immunological and clinical activity was observed. Adverse events were correlated with IFN-γ induction and dose of NHS-IL12. Increases in serum IL-10 levels, and tumor-infiltrating CD8^+^ T cells, and decreases in PBMC CD4^+^ populations after NHS-IL12 treatment were linked to exposure and may be evaluable as biomarkers in the future.

A strong genetic similarity exists between canine and human IL-12, with 84% homology for the ligand and 68% for the receptor (www.ensembl.org). NHS-huIL12 was used for this study since in-vitro work proved the NHS-huIL12 molecule is active in stimulating IFN-γ production from canine PBMCs (data not shown). Human IL-12 binds to the canine IL-12 receptor and invokes an IL-12 mediated response (data not shown). Additionally, earlier studies of nebulized IL-2 liposomes demonstrated regression of pulmonary metastases and increased bronchial lymphocyte and lytic effector cell activity in dogs with cancer [24]. These genetic, functional, and clinical data supported the use of comparative models to evaluate such cytokine-based therapies.

Results of this study suggest that there is a narrow therapeutic window for NHS-IL12 administration in clinical cancer patients. Doses of 0.8 mg/m^2^ were well tolerated, while dose of 1.6 mg/m² and 2.4 mg/m² resulted in Grade 4 or 5 adverse events. Fever, anemia, thrombocytopenia, lethargy and poor appetite were the most consistent Grade 4 or 5 events, with clinical evidence of cytokine-induced acute vasculitis and subsequent peripheral edema with pleural effusion. Peritoneal hemorrhage, likely secondary to severe thrombocytopenia and clinical evidence of disseminated coagulopathy, was also noted in one patient receiving 2.4 mg/m^2^.

Elevated serum IFN-γ levels (> 100 pg/ml) in dogs treated with 1.6 mg/m^2^ or higher were associated with toxicities. However, maximal IFN-γ induction was not directly linked to study deaths. Drug-related, high-grade, and life-threatening adverse events resulted from systemic vasculitis and/or SIRS, which are events commonly associated with systemic cytokine therapy. Additional escalation to cohorts of 1.0 mg/m^2^ or 1.2 mg/m^2^ may have helped to further refine tolerable doses of NHS-IL12 in tumor-bearing dogs and to best guide dose selection for first-in-human trials. Mining prospective evidence to identify patients who will experience severe adverse events should be the focus of continued efforts.

Consistent biomarkers of NHS-IL12 exposure were identified. In all but one dogs, a persistent elevation in serum IL-10 was detected after treatment. IFN-γ peaked consistently at 24 hours post-treatment and IL-10 was induced at 48 hours post-treatment. IL-10 levels remained elevated until or beyond 196 hours, which was the last time point analyzed. This is in keeping with previous preclinical and clinical reports that have described a persistent upregulation of IL-10 following treatment with IL-12; the induction of IL-10 has been implicated as a mechanism of IL-12 desensitization and attenuation of clinical activity[25,26]. Contribution of the immunoregulatory enzyme, indoleamine 2,3-dioxygenase (IDO) via tryptophan depletion or induction of immunomodulating tryptophan metabolites, should also be considered as a source of immunologic tolerance [27]. Clinical dosing intervals that allow IL-10 levels to return to baseline may optimize the clinical activity of NHS-IL12. Immunohistochemical analysis of tumor biopsies revealed an overall trend towards increased CD8^+^ T-cell infiltration following treatment with NHS-IL12, possibly the result of IL-12-induced T-cell proliferation [28,29]. Stimulation of anti-tumor immune responses mediated by CD8^+^ T cells may be a key mechanism of action through which NHS-IL12 could slow tumor growth.

NHS-IL12 can bind areas of tumor necrosis and thus the extent of necrosis within tumor, along with overall tumor volume, may potentially influence the PK profile of NHS-Il-12. Tumor biospies from dogs in the current study were collected most frequently using a Tru-Cut technique and thus, the physical size of the biopsies was likely not representative of the global extent of necrosis within tumors. A non-invasive imaging method that can provide a comprehensive quantitative assessment of both tumor volume and cell viability within solid tumors, such as ^18^F-fluorodeoxyglucose Positron Emission Tomography/Computed Tomography (^18^FDG-PET/CT), could be considered for future studies with these correlations in mind.

Although the overall objective response rate was low in this exploratory study, there were promising signs of therapeutic activity. In one dog (patient 0201) a 4.4 cm tumor was observed to completely regress. This response was characterized as a PR rather than a CR because computed tomography (CT) imaging to define maxillary bone involvement was not performed prior to treatment. Nine cycles of NHS-IL12 were given but this dog eventually succumbed to progressive disease in the sinus cavity 11 months after treatment initiation. Interestingly, serum NHS-IL12 levels were not detected at any pharmacokinetics time point, but measurable anti-drug antibodies indicated that exposure had occurred. Retreatment resulted in dramatic increases in cytokine and chemokine levels (GM-CSF, IL-2, IL-7, IL-15, IL-18, MCP-1). All but IL-18 are T-cell growth factors, but neither IL-7 nor IL-15 are produced by T cells. It is possible that IL-12 treatment induced expression of these cytokines by antigen-presenting cells and stroma. Predicting response in this patient was not possible, therefore methods to enhance NHS-IL12 directed targeting and to improve its reliability should be explored in future studies.

This study described the acute and long-term safety of NHS-IL12 administered subcutaneously and established a model for future evaluation of targeted immunocytokines. Immunotherapy’s best potential to improve outcome is challenged by Phase III standard of care requirements and protracted response monitoring needed to measure its effect. Comparative oncology studies can be conducted upfront in macroscopic and minimal residual disease settings and may better identify the therapeutic utility of these approaches. Furthermore, comparative efforts may improve the reliability of activity and safety biomarkers for human patients. Results from this study have been used together with other preclinical data to inform the design of a Phase I clinical trial of NHS-IL12 in human cancer patients (NCT01417546).

## Methods

### Comparative Oncology Trials Consortium

The goals and infrastructure of the COTC have been described previously [30–32]. All COTC trial data are reported electronically and reviewed contemporaneously through the Cancer Central Clinical Database (C3D) modified for use in canine clinical trials. Trials are centrally managed and directed through the NCI-Comparative Oncology Program (COP).

### Trial eligibility and enrollment

Client-owned pet dogs with histologically confirmed melanoma, favorable performance status (modified Eastern Cooperative Oncology Group [ECOG] Performance Status grade 0 or 1), and informed owner consent were eligible for enrollment. Eligibility criteria required a newly diagnosed or recurrent melanoma, where the measureable tumor was amenable to serial biopsy and > 3 cm but < 8 cm in the longest diameter. Physical examination and laboratory assessments, including complete blood count (CBC), serum biochemical profile, and urinalysis (UA), were performed to evaluate eligibility prior to enrollment. Exclusion criteria removed dogs weighing less than 10 kg, those with significant co-morbidities (such as renal, liver, and heart failure or coagulopathy), history of immune-mediated disease, or concurrent chemotherapy, radiation therapy, or biological therapy. Tumor staging included thoracic radiographs performed prior to study initiation. All dogs were evaluated uniformly and treated within a defined clinical protocol with Institutional Animal Care and Use Committee (IACUC) approval at each COTC enrollment site (University of California-Davis, Colorado State University, University of Tennessee, University of Missouri, Texas A&M University, University of Wisconsin-Madison, and Tufts University). The NCI-COP reviewed the eligibility screening and approved trial entry of each dog.

### Treatment, monitoring and safety assessments

Dogs underwent a complete physical examination, laboratory assessments (CBC, serum biochemical profile, coagulation panel, UA, whole blood, PBMCs, and serum collections), and pre-treatment biopsies at enrollment. Vital signs (core temperature, pulse, respiratory rate) were recorded at baseline. Dogs received NHS-IL12 subcutaneously on day 1 according to their dosing cohort. Dogs remained hospitalized for serial pharmacokinetic analysis, serum IFN-γ/cytokine and PBMC collections, and temperature measurements over the first 48 hours. Dogs were discharged into the care of their owners after the 48-hour blood draw. Owners were required to monitor their dog’s temperature once daily for 5 days after NHS-IL12 administration. Additionally, owners completed an *Owner Assessment Form* at baseline (day 0), then weekly, to record impressions of their dog’s clinical status throughout the study period.

A primary study objective was to define acute and chronic toxicities associated with the subcutaneous administration of NHS-IL12. Expected adverse events were outlined in the study protocol. Repeat laboratory assessments (CBC, biochemical profile, coagulation panel, and UA) were performed on days 8, 15, and 29 to define safety. All bloodwork was performed uniformly at a laboratory adhering to GLP (Antech Diagnostic Laboratories, Irvine, California). VCOG-CTCAE v. 1.0 were used to define DLTs [21]. DLTs were defined as any grade 3 or 4 hematologic or non-hematologic toxicities. Transient (< 24 hours) grade 3 or 4 neutropenia, thrombocytopenia, or fever associated with grade 2 or less lethargy/fatigue, or transient grade 3 increases in hepatic enzymes (alanine aminotransaminase, aspartate aminotransaminase up to > 10x upper limit of normal [ULN] and/or alkaline phosphatase up to > 20x ULN) or bilirubin (up to > 5x ULN) that trend downward to grade 2 or better by day 21 were prospectively defined in the study protocol as expected and non-dose limiting.

Dose-limiting adverse events guided the rapid dose-escalation study plans. Grade 2 toxicity in 1/1 dog triggered enrollment of an additional two dogs at that prescribed dose. DLT toxicity in 1/3 dogs in a cohort necessitated cohort expansion (up to six dogs) to ensure tolerability. The maximum tolerated dose was defined as one dose level below the maximum achieved in the dose-escalation phase. Any and all adverse events were collected within the electronic database reporting system (C3D) following strict one-week reporting timelines.

### Biological collections

Whole blood, PBMCs, and serum were collected for all dogs. Pre- and post-treatment PBMCs were collected for T-cell subset analysis on days 1, 2, 3, and 8. An eight-point pharmacokinetic analysis of serum NHS-IL12, IFN-γ, and cytokine levels was performed pre-treatment (0 minutes), and at 1 hour, 2 hours, 4 hours, 8 hours, 24 hours, 32 hours, and 48 hours (prior to hospital discharge on day 3) after drug administration. Subsequent serum collections were made on days 8, 15, 22, and 29. PBMCs were shipped the day of collection, while serum samples were frozen and stored at -80°C until shipping. Samples collected up to and including day 8 were mailed at the end of a dog’s first week on the study and all other samples were shipped at the end of the study period.

### Tumor biopsies

Dogs underwent a biopsy prior to receiving NHS-IL12 to establish a baseline for future evaluations. Pre-treatment biopsies were incisional in nature and guided by the location of the tumor. Keyes 6 mm punch biopsy instruments, 14g Tru-cut needle, and open biopsy techniques were acceptable. Two pre-treatment samples were obtained at differing planes/angles within the tumor. Each sample was sectioned, placed in formalin, RNAlater, and flash frozen. Similarly, two post-treatment samples were obtained at differing planes/angles within the tumor on both days 8 and 29 and processed as described above. Histopathologic analysis of tumor samples is described below. Samples were stored at room temperature or -80°C respectively and shipped at patient study completion for analysis.

### Lymph-node fine-needle aspirates

Aspirates of regional lymph nodes draining the primary target lesion were performed when applicable. Aspirates were collected on day 1 (pre-treatment), and on days 8 and 29 for flow cytometry analysis to define subset populations of immune cells. Lymphoid populations were characterized using dendritic and T- and B-cell markers: CD3, CD4, CD8, CD21, MHCII, CD32, CD86, Treg/FoxP3, CD11c, CD40, and CD1a as described below.

### Response assessment

Dogs that completed the 29-day study were evaluable for response. Clinical response at day 29 was based on RECIST criteria [22,23]. Dogs with PD or SD at day 29 were deemed off-study. Dogs with resectable tumors at day 29 could undergo definitive resection, although this would render them off-study. In the case of a measured response (PR or CR), dogs were evaluated monthly until PD was documented or other treatment was administered. Dogs were eligible to continue receiving NHS-IL12 beyond day 29 (dogs with a PR or CR only) on an individual patient basis. If PD was documented at any point during the study, even if prior to day 29, the dog was removed from the study and permitted to receive other treatment according to the recommendations of their COTC site principal investigator.

### NHS-IL12 structure and formulation

EMD-Serono generated a fusion protein (i.e. NHS-IL12), by transfection of Ab-p35 subunit fusion constructs at the C-terminus into an NS/0 LD cell line already engineered to express high levels of the p40 subunit of human IL-12 [33]. During the course of this study, 3 lots of NHS-IL12 were administered to dogs (Non-GMP Lots # T07-009-12 and T09-001-3; GMP engineering lot 1510A01). Lot number and date of use was recorded for all dogs.

### NHS-IL12 subcutaneous administration

NHS-IL12 was administered subcutaneously on day 1. Dogs were eligible for retreatment every 28 days if they achieved an objective response by RECIST criteria. The dose was determined by the NCI-COP based on an individual dog’s weight (converted into body surface area [m^2^]) and study cohort where applicable (0.4 mg/m^2^; 0.8 mg/m²; 1.6 mg/m²; 2.4 mg/m²). The total prescribed volume (1 ml) was delivered subcutaneously within the dorsal skin fold for all dogs. Volume was standardized for all dogs and diluent added to each dosage as needed. A 22-gauge needle was used for drug administration and the entire contents were dispelled prior to removing the needle from the subcutaneous space.

### ELISA detection of serum NHS-IL12 levels for pharmacokinetics analysis

For the detection of NHS-IL12 in serum samples, a commercially available human IL-12 ELISA kit (R&D Systems/Bio-Techne, Minneapolis, Minnesota) was used and further adapted (calibration range: 31.3 to 2,000 pg/ml on plate; lower limit of quantitation in samples: 0.62 ng/ml). The kit includes microplates pre-coated with an anti-human IL-12 antibody. Serial dilutions of NHS-IL12 protein were used as a calibration standard. Serum samples (run in duplicate) were incubated in the assay plate for 2 hours before being washed and incubated with a horseradish peroxidase-conjugated anti-IL-12 detection antibody. After another wash, tetramethylbenzadine (TMB) chromogenic substrate was added. The reaction was stopped by addition of an acid solution, and the optical density of each sample in the assay plate was determined using a microplate reader set at 450 nm. The concentration of NHS-IL12 in each serum sample was calculated from a standard curve generated from a 4-parameter logistic curve-fit. The assay was not evaluated for cross-reactivity to canine IL-12.

### Immunogenicity analysis

Label-free surface plasmon resonance technology (Biacore Lifesciences/GE Healthcare Bio-Sciences, Pittsburgh, Pennsylvania) was used to measure auto-antibody production to NHS-IL12. For this purpose, CM5 Series S chips were coupled with either NHS-IL12 or purified human IgG (Jackson Immuno Research, West Grove, Pennsylvania) for the detection of anti NHS-IL12 antibodies. For analysis of the samples taken at later stages of the study course (patients 0101, 0301, 0102, 1001, 0104, 0302, 0103, 0303, 1902, 1004, 0502, 0201), CM5 chips were additionally coated with the p40 subunit of human IL-12 and the p70 dimeric recombinant IL-12 in order to assess the immunogenic potential of the cytokine subunits of NHS-IL12. To avoid non-specific serum protein interaction with the chip surface, each of the serum samples was diluted with running buffer containing 1 mg/ml carboxymethyl dextran before analysis. All test samples were injected over the chip surface and by subtracting the surface signal after the sample injection from the surface signal prior to the sample injection, a binding signal for that sample was calculated. No calibration curve was generated. Instead, the pre-treatment samples taken from each dog were used as control samples to demonstrate positive binding signals. Antibodies to cytokines are routinely detected in both human and dog studies [34]. These antibodies are expected to be neutralizing after 30–45 days of exposure.

### ELISA detection of serum IFN-γ levels

IFN-γ in serum samples was detected using a commercially available canine IFN-γ ELISA kit used according to the manufacturer’s protocol (R&D Systems, Cat# CAIF00). The kit includes microplates pre-coated with an antibody specific for canine IFN-γ. A standard curve was created using a recombinant canine IFN-γ standard. Serum samples (run in triplicate), and a canine IFN-γ positive control, were incubated in the assay plate for 2 hours before being washed and incubated with a canine-specific anti-IFN-γ-biotin conjugate. Following another wash, detection was made by addition of a streptavidin-horse radish peroxidase conjugate, and, after a final wash, TMB chromogenic substrate was added. The reaction was stopped by addition of an acid solution, and the optical density of each sample in the assay plate was determined using a microplate reader set at 450 nm. The concentration of IFN-γ in each serum sample was calculated from a standard curve generated from a 4-parameter logistic curve fit.

### Serum cytokine profiling

Serum cytokine panels quantified levels of IL-2, IL-4, IL-6, IL-7, IL-8, IL-10, IL-15, IL-18, GM-CSF, IFN-γ, IP-10, KC, MCP-1, TNF-α using the CCYTOMAG-90K canine cytokine/chemokine kit from Millipore (Billerica, Massachusetts). Samples were prepared according to the manufacturer’s instructions, with the exception of the following modifications: a) the volume of all reagents used was halved, while keeping the concentration standard after validating this approach by comparison with the recommended volume; and b) the highest concentration of the standard curve was eliminated after the first 4 samples, since no dog’s cytokine/chemokine levels approached this highest value. All samples were analyzed using a Bio-Plex 200 multiplex assay system (Bio-Rad, Hercules, California).

### Flow cytometry of PMBCs and regional lymph node samples

Blood was received on ice in EDTA blood collection tubes and analyzed as soon as received. Red blood cells were lysed using a high salt solution consisting of 0.0005% phenol red, 150 mM ammonium chloride, 10 mM potassium bicarbonate, and 0.1 mM EDTA (reagents from Sigma, St. Louis, Missouri). PBMC or lymphocytes were added at a concentration of 5 x 10^5^ to 1 x 10^6^ per well in 96-well round bottom plates. Lymph node aspirates were washed once in FACS buffer (PBS with 2% fetal bovine serum and 0.1% sodium azide) before being transferred to round bottom 96-well plates. The cells were then centrifuged and washed using FACS buffer, and cell pellets were resuspended in 10µl/well of blocking agent (normal canine serum, 16 µl/ml human IgG, mouse anti-FCR III, and 0.05% sodium azide (all from Jackson Immunoresearch). Antibody combinations for flow cytometric analysis of T cells, B cells, and dendritic cells were added in a 40 µl total volume as described below.

### B-cell panel for lymph node aspirates and white blood cells collected from blood

1:10 mouse anti-canine CD21-PE (clone CA2.1D6; AbDSerotec, Raleigh, North Carolina) and 1:10 rat anti-canine MHC class II-FITC (clone YKIX334.2; AbDSerotec).

### Dendritic-cell panel for lymph-node aspirates

1:1 mouse anti-canine CD11c primary antibody (clone CA11.6A1; AbDSerotec), secondary antibody 1:200 F(ab)2 fragment donkey anti-mouse IgG-PE (Jackson Immunoresearch), and 1:10 rat anti-canine MHC class II-FITC (clone YKIX334.2; AbDSerotec).

### T-cell panel for lymph node aspirates and white blood cells collected from blood

1:200 rat anti-canine CD8-Alexa 647 (clone YCATE55.9; AbDSerotec) and 1:200 rat anti-canine CD4-FITC (clone YKIX302.9; AbDSerotec).

Primary stains were incubated at room temperature for 30 minutes before being washed twice in FACS buffer. Secondary antibodies were also incubated for 30 minutes at room temperature in the case of the dendritic cell panel. T-cell panels were fixed and permeabilized overnight in fixation/permeabilization buffer (eBioscience, San Diego, California). The following day, T-cell panels were washed twice with 1X permeabilization buffer (eBioscience) diluted in distilled water before being stained with 1:200 anti-mouse/rat Foxp3-PE (clone FJK16a; eBioscience) for 30 minutes. A directly conjugated rat IgG_2A_ antibody was used as the isotype control. B-cell and dendritic-cell panels were fixed using a 1% solution of paraformaldehyde for 20 minutes. All cells were washed an additional two times before being resuspended in FACS buffer in cluster tubes ready for analysis by flow cytometry. Flow cytometry was carried out on a Cyan flow cytometer (DakoCytomation/Beckman Coulter, Fort Collins, Colorado) and Summit software for data analysis (DakoCytomation). Approximately 100,000 events were analyzed for each sample. Analysis gates were set on the live lymphocyte population based on typical forward- and side-scatter characteristics. The percentage of Tregs was calculated by determining the percentage of FoxP3^+^CD4^+^ cells within the CD4^+^ T-cell population. The percentages of B cells, CD4^+^ and CD8^+^ T cells, and dendritic cells were also determined.

### Histopathology and immunohistochemistry methodology

Hematoxylin and eosin (H&E) histologic analysis plus semi-quantitative scoring of necrosis and extent/character of inflammatory infiltrates were performed for all tumor samples (pre-treatment, days 8 and 29, and every 28 days thereafter, if appropriate). Immunohistochemistry including CD3, CD4, CD8a, CD79a, CD18, and IL-12 plus photo documentation and semi-quantitative scoring across the same study samples was also performed. Antibodies used for immunohistochemistry are described below.

### B-cell immunohistochemistry

Mouse monoclonal anti-human CD79a (1:125 dilution; Clone HM57, Dako Inc., Carpinteria, California).

### T-cell immunohistochemistry

Rat monoclonal anti-canine CD3e (1:20 dilution; Clone CD3-12; P. Moore, UC Davis, California); mouse monoclonal anti-canine CD4 (1:10 dilution; Clone CA13.1E4; P. Moore, UC Davis, California); mouse monoclonal anti-canine CD8 (1:10 dilution; Clone CA9.JD3; P. Moore, UC Davis, California).

### Beta-integrin (monocyte) immunohistochemistry

Mouse monoclonal anti-canine CD18 (1:10 dilution; Clone CA16.3C10; P. Moore, UC Davis, California).

Serial sections from paraffin tissue blocks from each of the submitted formalin-fixed samples were deparaffinized, and rehydrated for routine immunohistochemical labeling. Immunohistochemical labeling was used to detect expression of CD18, CD3e, CD79a, and IL12RB2. Deparaffinization, immunohistochemical labeling, and counterstaining was performed on the Bond maX Automated Staining System (Vision BioSystems, Leica, Bannockburn, Illinois) using the Bond Polymer Detection System (Vision BioSystems, Leica, Bannockburn, Illinois). Antigen retrieval for CD3e and CD79a was done on the Bond maX Automated Staining System by incubating slides with retrieval solution ER1 (Vision BioSystems, Leica, Bannockburn, Illinois) for 20 minutes. Antigen retrieval for CD18 and IL12RB2 was achieved by incubation of slides in antigen retrieval solution (Dako Inc, Carpinteria, California) in a steamer (Black & Decker, New Britain, Connecticut) for 20 minutes. Primary antibodies and their dilutions, source, and host are listed above. The immunoreaction was visualized with 3,3-diaminobenzidine substrate (Vision BioSystems, Leica, Bannockburn, Illinois) and sections were counterstained with hematoxylin. Positive immunohistochemical controls included a normal canine lymph node to which the appropriate anti-sera were added. For negative controls, the primary antibodies were replaced with homologous non-immune sera. Only cytoplasmic/membrane-associated labeling within cells was evaluated as positive. Additional serial sections of each sample were stained with H&E according to routine protocols.

Serial sections of frozen tissue were cut on a Vibratome cryostat. Following fixation in cold acetone (-20^°^C) for 3 minutes, immunohistochemical labeling for CD4 and CD8 was performed on the Bench Mark Automated Staining System (Ventana Medical Systems, Inc., Phoenix, Arizona) using the Ultra V-Red Detection (Alkaline Phosphatase Red) System with a universal protein blocker (Dako Inc., Carpinteria, California). Sections were counterstained with hematoxylin. Positive immunohistochemical controls included a normal canine lymph node to which the appropriate anti-sera were added. For negative controls, the primary antibodies were replaced with homologous non-immune sera. Only cytoplasmic/membrane-associated labeling within cells was evaluated as positive. Additional serial sections of each sample were stained with H&E eosin according to routine protocols.

### CD8 Grading Scale

0 = no CD8^+^ cells; 1 = less than 10% of inflammatory cells are CD8^+^; 2 = 10–50% of inflammatory cells are CD8^+^; 3 = more than 50% of inflammatory cells are CD8^+^.

## Supporting Information

S1 File The ARRIVE Guidelines Checklist. (DOC)
